# Evaluation of the tool “Reg Refine” for user‐guided deformable image registration

**DOI:** 10.1120/jacmp.v17i3.6025

**Published:** 2016-05-08

**Authors:** Perry B. Johnson, Kyle R. Padgett, Kuan L. Chen, Nesrin Dogan

**Affiliations:** ^1^ Department of Radiation Oncology Miller School of Medicine, University of Miami Miami FL USA; ^2^ Department of Radiation Oncology Willis‐Knighton Cancer Center Shreveport LA USA

**Keywords:** deformable image registration, Reg Refine, registration validation, commercial registration systems, CBCT noise

## Abstract

“Reg Refine” is a tool available in the MIM Maestro v6.4.5 platform (www.mimsoftware.com) that allows the user to actively participate in the deformable image registration process. The purpose of this work was to evaluate the efficacy of this tool and investigate strategies for how to apply it effectively. This was done by performing DIR on two publicly available ground‐truth models, the Pixel‐based Breathing Thorax Model (POPI) for lung, and the Deformable Image Registration Evaluation Project (DIREP) for head and neck. Image noise matched in both magnitude and texture to clinical CBCT scans was also added to each model to simulate the use case of CBCT–CT alignment. For lung, the results showed Reg Refine effective at improving registration accuracy when controlled by an expert user within the context of large lung deformation. CBCT noise was also shown to have no effect on DIR performance while using the MIM algorithm for this site. For head and neck, the results showed CBCT noise to have a large effect on the accuracy of registration, specifically for low‐contrast structures such as the brainstem and parotid glands. In these cases, the Reg Refine tool was able to improve the registration accuracy when controlled by an expert user. Several strategies for how to achieve these results have been outlined to assist other users and provide feedback for developers of similar tools.

PACS number(s): 87.44.Qr, 87.57.nj, 87.57.c

## I. INTRODUCTION

Deformable image registration (DIR) is the nonaffine process of mapping voxels from one image to the next where the individual vectors which describe the mapping may vary in both magnitude and direction from their neighbors. The algorithms which allow this process to occur are complex, diverse and, more often than not, obscured to the end user, particularly those commercially available to the radiation oncology clinic. In implementing one of these algorithms within the context of a commercial platform, two important questions to consider are: 1) How to quantify the accuracy of the system? and 2) How to best utilize the system for a given use case?

The former question has been an area of on‐going research for the past several years and the subject of an upcoming report by AAPM Task Group 132.^(1)^ The framework for validation relies upon the establishment a ground‐truth deformation. This takes several forms, including physical phantoms which can be deformed mechanically in known ways,^2^, ^3^ real patient images with identifiable landmarks which can be tracked between images,^4^, ^5^ and digital phantoms which can be deformed manually to create a linked pair of reference images.^(6)^ While extensive time and effort is required to establish a ground‐ truth deformation using these methods, several authors have graciously performed this work and made such data publicly available. Examples include the Point‐validated Pixel‐based Breathing Thorax Model (POPI) for lung,^(4)^ the DIR‐Lab Thoracic 4D CT model also for lung,^(5)^ and the Deformable Image Registration Evaluation Project (DIREP) for head and neck.^(6)^ These datasets are a great option for many clinics that may not have the capital to invest in validation‐specific software such as ImSimQA (Oncology Systems Limited, Shrewsbury, UK) or the resources to create their own phantoms. Additionally, because the datasets are meant to be widely distributed, comparisons can be made between software, algorithms, and institutions all using a standardized baseline. One limitations of these datasets, however, is that the primary and secondary images used to define the ground truth exist only as a single modality — fan‐beam CT.

With the horizon for adaptive planning rapidly approaching, there is a need to further asses the performance of deformable algorithms in situations in which a cone‐beam CT (CBCT) would be adapted to a planning CT. One aspect of this deformation which has the potential to affect accuracy is the increased noise of CBCT images. A limited number of studies have investigated this sensitivity, with the results being mixed. In one study, quantum noise as found in 4D CT was found to alter the accuracy of DIR in the lung.^(7)^ Four different DIR algorithms were assessed, and changes from the baseline DIR was noticed even when the noise level was low. In a separate study utilizing a B‐spline algorithm, the authors concluded that the noise level of CBCTs did not reduce the accuracy of DIR for the use case of prostate, bladder, and rectum contour propagation.^(8)^ This result was confirmed in a separate study which found differences in noise sensitivity between B‐spline and Demons algorithms in low‐contrast scenarios.^(9)^ In addition to the choice of algorithm, the studies also differed in the way in which noise was added to CT data to create pseudo‐CBCT/4D CT images. In the first study, Gaussian noise was added to simulating different noise magnitudes. In the second two studies, the noise power spectrum (NPS) was utilized to simulate different noise magnitudes while also attempting to reproduce a realistic noise texture. This latter method is expanded upon in this work to include more clinically relevant spectrums, and to provide a comparison between different anatomical regions and how they differ in their sensitivity to CBCT noise.

Beyond assessing the baseline accuracy of a DIR algorithm for different use cases, it is also important to know how best to utilize the available tools within a given software system to achieve the optimal result. MIM Maestro v6.4.5 (MIM Software, Cleveland, OH) is a commercial software system widely used for deformable image registration. A unique feature of MIM is that it provides a form of user guidance over the deformation process through utilization of the Reg Refine tool. This tool allows the user to visually asses the deformable registration between two points and to correct the registration if needed by applying either manual or local box‐based rigid alignments. The specific alignment between two points can then be “locked” before a repeat DIR is run. The use of the term “locked” is meant to describe the fact that the alignment between the two points is provided to the DIR algorithm as new initial conditions. The number of locks is limited only by computer memory, and repeat DIR can be run as many times as desired. Currently there is no literature available which describes how to best utilize the Reg Refine tool. Additionally, there have been no published studies which quantify the results of using the Reg Refine to improve registration accuracy.

In this work, these questions and those related to CBCT noise are addressed for two different anatomical regions. For the case of lung cancer, the freely available POPI model was used to provide a ground‐truth deformation. For the case of head and neck cancer, the downloadable phantoms of the Deformable Image Registration Evaluation Project were utilized. A heuristic approach was taken whereby several deformations were run while varying the location and quantity of Reg Refine locks. For a separate subset of cases, realistic CBCT noise was also simulated and added to the datasets. The effect of this addition was tested using different DIR approaches available within the MIM Maestro software.

## II. MATERIALS AND METHODS

### A. Algorithm

Like most commercial systems, MIM Maestro v6.4.5 incorporates a proprietary DIR algorithm. Based on previous details released by the manufacturer it is known that the algorithm involves a freeform (Demons‐based) transformation which is applied through a multiresolution approach.^(10)^ The algorithm begins by accounting for gross differences using a course grid. The resolution is then increased and local changes are address over a small scale. The final resolution has a maximum grid size of $3\times 3\times 3\;{\text{mm}}^{3}$. An intensity based sum of square difference approach is used to assess the goodness of match, and a strategy based on gradient descent is used for optimization. While the algorithm does not ensure a positive Jacobian, multiple types of regularization are used to keep the transformation reasonable. It is noted that the algorithm actively attempts to match bone and avoid tears/folds in the deformation field.^(11)^ Once a registration is defined, both contours and dose can be propagated.

Reg Refine is a tool available with MIM Maestro which provides visual assessment and user guidance of the deformable registration in the following manner:

1. Following an initial DIR, a fusion window is opened where the user may fade between primary and secondary datasets within a given rectangular space.
2. At this point the two datasets are linked via a rigid registration (i.e., all the vectors pointing from one dataset to the next are identical).
3. The magnitude and direction of these vectors are defined specifically by the voxel at the center of the window and its link to the secondary dataset. This link was previously established during the initial DIR.
4. The window is moveable, and thus as the user shifts the fusion window, the rigid registration changes based on the deformation vector found at the center voxel.
5. Because the user is able to fade between the datasets, it is then possible to “visualize” the deformation vector of the center voxel as interpreted by the rigid registration applied to the entire dataset.
6. The user may alter the registration either through manual or box‐based rigid registrations. For a box‐based registration there is a second registration window that appears within the fusion window. The size of this window can be changed. Only the voxel intensities found within it are used during the rigid registration, which is based on a sum of square difference minimization.
7. Once the registration for the center voxel has been finalized, the link between the primary and secondary datasets at this point can be “locked”. The lock effectively replaces the deformation vector in the initial DVF with the new deformation vector defined during the user guided registration.
8. Finally, the modified DVF is utilized by the deformable algorithm as improved initial conditions during a second deformable registration in order to achieve a result closer to that defined by the user.

### B. Ground‐truth models

In this work, two different types of ground‐truth validations were utilized. For lung cancer, the POPI model includes six 4D CT datasets (POPI_1, POPI_2, etc.) where 100+ lung bifurcations have been identified on each phase of breathing by medical experts. During DIR as performed in this study, the points are propagated from the end of inhalation CT (Phase 0) to the end of exhalation CT (Phase 50). The difference in location between the propagated points and those previously identified on the end of exhalation CT determine the registration error.

For head and neck cancer, the Deformable Image Registration Evaluation Project (DIREP) provides 10 virtual patient datasets (DIREP_1, DIREP_2, etc.). Included for each patient are a start of treatment CT (SOT), end of treatment CT (EOT), and the ground‐truth deformation vector field (DVF). As described more completely in Pukala et al.,^(6)^ the ground‐truth DVF was determined through manual, forward‐based deformation using the software ImSimQA. The software allows the user to manipulate a source dataset to match a target dataset using a biomechanical algorithm in combination with human‐guided thin plate splines. This allows for the modeling of head rotation and translation, mandible rotation, shoulder movement, spine flexion, hyoid movement, tumor/node/OAR shrinkage, and weight loss. In this work DIR was performed in the EOT to SOT direction. The results were analyzed by comparing the ground‐truth DVF with the DVF provided by each deformable registration.

### C. CBCT noise

**Figure 1:**
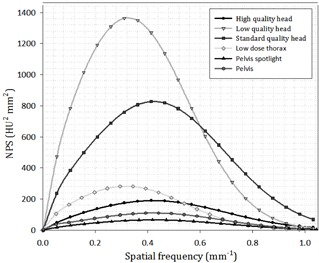
Noise power spectrum (1D profile) for all CBCT protocols available for the Varian Trilogy OBI v1.6 system.

Image noise can be described in terms of both magnitude and texture. Noise magnitude refers to the amount of variation in pixel intensity across a uniform ROI and is commonly quantified using the statistical measure of standard deviation. Noise texture, on the other hand, deals with spatial frequency and differentiates noise appearance as either grainy or smooth. Images created using a CT or CBCT modality will share a similar form of noise texture due to the type of filtering applied during image reconstruction. This can be seen in the noise power spectrum which plots noise power $\left({\text{HU}}^{2}{\text{mm}}^{2}\right)$ versus spatial frequency $\left({\text{mm}}^{-1}\right)$ (Fig. 1). The NPS was previously adapted for CT imaging through a specific method of data collection and analysis.^12^, ^13^ The method relies upon the scanning of a uniform phantom and the selection of several ROIs at an equal radius from the center of a given image. The ROIs are detrended, and the 2D Fast Fourier Transform is applied to each subvolume. Data averaging is applied across all ROI's within a given image and also over several CT slices. The final result should be radially symmetric (approximately) and have the appearance of a torus in frequency space. Due to the symmetry of the data, a radial profile across the torus provides a convenient way to express the NPS as a 1D plot. The integral of the NPS is equal to the variance, or equivalently the noise magnitude squared. CBCT protocols with lower exposures (mAs) will have larger NPS curves because they are inherently more noisy (compare “Low Quality Head” with High Quality Head” in Fig. 1).

For this work two different phantoms were used for data acquisition. In the first scans, the uniform region (CTP486) of a Catphan 500 phantom was scanned using each of the three predefined CBCT protocols available on the Varian Trilogy On‐Board Imaging v1.6 (Varian Medical Systems, Palo Alto, CA) system designated for head patients. In each scan, the phantom was aligned at isocenter and scanned twice in order to facilitate image subtraction during the detrending process. In the second acquisition set, the Varian uniform body phantom was scanned using each of the three predefined CBCT protocols available for body patients. Again, each scan was repeated twice to facilitated image subtraction. A full listing of the parameters for each scan can be found in Table 1.

In order to create 1D NPS profiles, each dataset was processed using MATLAB vR2014b (MathWorks, Natick, MA) and the previously discussed method. Roughly 20 slices were included from each scan, and 16 ROIs were selected from each slice. The size of each ROI was 40 pixels to a side, and the ROIs were chosen along a circumference swept by a radius 80 pixels in length. Detrending of each ROI was investigated using both image subtraction and through the use of a custom 2D detrending script written in MATLAB. Both methods provided similar results, therefore the custom 2D detrending script was used to process the data.

Once a 1D NPS profile was measured for each CBCT protocol, the data was fit using the form shown in Eq. (1). This form includes the effects of ramp‐up and roll‐off which relate to the filtering of 1/r blurring and high frequency content during image reconstruction. Parameters $a_{1}–a_{4}$ are fitting parameters, where $a_{1}$ describes the height of the peak, $a_{2}$ determines the position of the center, $a_{3}$ controls the width of the bell shape, and $a_{4}$ determines the speed of the ramp‐up. The independent parameter, x, represents spatial frequency, and the dependent parameter, f(x), represents the NPS for a given frequency.

(1) $$f(x)=a_{1}e^{-{\left(\frac{x-a_{2}}{a_{3}}\right)}^{2}}(1-e^{-a_{4}x})$$

The fitting of the data helped smooth the profiles and parameterize the data whereby CBCT noise could be recreated without the collecting of actual scanned data. Once the data were smoothed, a custom MATLAB script was used to convert the 1D NPS profile into a 2D NPS by relying on the symmetry of the NPS profile to fill the a 2D matrix. The inverse FFT was then applied to transition from the frequency domain to the spatial domain, allowing for the creation of a pure noise image. Pseudo‐CBCT datasets were created for both the POPI and DIREP phantoms by adding the pure noise images to the secondary datasets from each phantom series. The secondary datasets for the POPI model were represented by the exhalation CT images and for the DIREP model by the end of treatment CT images.

**Table 1:** Parameters used during the collection of CBCT images on the Varian Trilogy OBI v1.6.

*Mode*	*High Quality Head*	*Standard Quality Head*	*Low Quality Head*	*Pelvis*	*Pelvis Spotlight*	*Low Dose Thorax*
FOV (cm)	24.3	24.3	24.3	25.6	24.0	25.6
Matrix (px)	512	512	512	512	512	512
Pixel size (mm)	0.475	0.475	0.475	0.500	0.469	0.500
Length (cm)	18	18	18	16	16	16
kVp	100	100	100	125	125	110
mA	80	20	10	80	80	20
Filter	Ram‐Lak	Ram‐Lak	Ram‐Lak	Ram‐Lak	Ram‐Lak	Ram‐Lak
Scan option	Sharp	Sharp	Standard	Sharp	Sharp	Standard
Exposure	726	145	72	676	718	260
Exposure time	9075	7260	7260	8450	8975	13000
Recon diameter	243	243	243	256	240	256

### D. Deformable registration

The first iterations of DIR were focused on assessing the baseline accuracy of the MIM algorithm. This was done by performing deformable image registration for all POPI and DIREP phantoms without the addition of CBCT noise or the use of the Reg Refine tool. The MIM software requires a rigid registration prior to any DIR. For head and neck phantoms, the SOT/EOT datasets were already well aligned, and thus the rigid registration was confirmed “as is”. For lung phantoms, the choice was made to focus the rigid alignment on either the apex or base of the lung. The effect of choosing one versus the other was examined during data analysis.

The second set of tests involved the original POPI and DIREP phantoms, but with the additional use of the Reg Refine tool. Registrations for the head and neck phantoms were performed by three separate users representing different levels of experience. User 1 had been recently introduced to DIR and the Reg Refine tool, User 2 was an advanced user with some clinical and research usage, while User 3 was an expert user with significant clinical and research usage. The intent of commissioning multiple people to perform registrations was two‐fold. The first purpose was to investigate whether user experience played a role in the accuracy of the DIR. The second purpose was to take a team approach in refining the use of the tool. Multiple users provided increased feedback as to which methods were successful and which were not.

For the POPI model, a single advanced user performed all registrations. For these cases, the number of Reg Refine locks was also guided by a sensitivity study performed using Phantom 2 of the POPI model (POPI_2). This phantom was chosen based on the mean displacement (difference between linked voxels on the primary and secondary datasets) being the largest amongst all similar phantoms. As detailed in the following section, the study indicated the usage of between 5 and 15 Reg Refine locks. These locks were positioned at a various locations during separate iterations of DIR. A heuristic approach was used to optimize placement.

In order to investigate the effect of CBCT noise on DIR accuracy, a third set of deformations were performed using the aforementioned pseudo‐CBCTs as the secondary or “floating” dataset. An initial study was performed using POPI_2 and DIREP_10 in which the selection of CBCT protocol was investigated. The protocol “low quality head” showed the largest effect during the deformable registration of DIREP_10 and was chosen for use with all head and neck phantoms. Noise appeared to have no effect on POPI_2. As a result, further testing of the lung phantoms was limited to just a few other cases which will be discussed in the following section.

### E. Evaluation

For the lung phantoms, error is defined by the difference in the location of each propagated point in comparison with their ground‐truth locations in the primary dataset. For the head and neck phantoms, there are three datasets to consider: the primary image (*SOT*), the secondary image (*EOT*), and the output of the deformation which is the deformed secondary image (*dEOT*). The ground‐truth DVF is defined as a vector field pointing from the primary to the secondary dataset $\left(\vec{GT}\right)$. A test DVF is defined as a vector field pointing from the deformed secondary dataset to the secondary dataset $\left(\vec{Test}\right)$. It is done this way to insure that each vector has a starting and ending point. The error reported in this study is thus the difference between the two vector fields (Δ), that is to say the difference in colocated points as mapped to the secondary dataset (see (2), (5)). Contouring masking allowed for the assessment of DIR error within specific structures. The structures included in this study were the brainstem, spinal cord, mandible, left parotid, and right parotid.

(2) $$dEOT=(x_{1},y_{1},z_{1}),\;SOT=(x_{1},y_{1},z_{1})$$

(3) $$EOT_{dEOT}=(x_{2},y_{2},z_{2}),\;EOT_{SOT}=(x_{3},y_{3},z_{3})$$

(4) $$\vec{Test}=EOT_{dEOT}-dEOT,\vec{GT}=EOT_{dEOT}-SOT$$

(5) $$\Delta =\vec{Test}-\vec{GT}$$

The statics used to quantify error included the mean, median, and max registration error. While the mean error is a more commonly reported statistic, the median error may be a more appropriate metric for this type of analysis where outliers have the potential to skew the data. Additionally, the percentage of voxels/points having an error less than 2 mm was also tracked. This was included after considering the preliminary recommendations of AAPM Task Group TG‐132 where the metric was mentioned with a proposed goal (as opposed to tolerance) of 95% for digital phantom test cases.^(1)^

## III. RESULTS

### A. Lung phantoms

**Figure 2:**
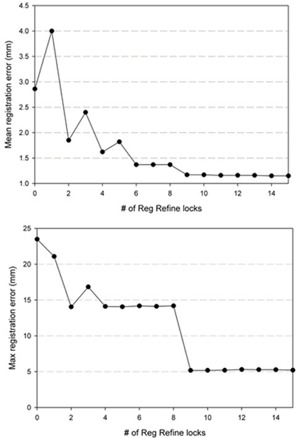
Mean and max registration error vs. the number of Reg Refine locks as measured for the POPI_2 phantom.

Results for deformable registrations involving the POPI model are shown in 2, 3. The data were separated into two tables by isolating the POPI_2 phantom. This was done due to the fact that POPI_2 had a mean displacement twice as large as the other phantoms (14 mm compared to 7 mm) and performed differently during DIR. As seen in Table 2, the error associated with this phantom was heavily dependent on the initial rigid registration being aligned to either the base or apex of the lung. While the base of lung registration performed noticeably better during the initial DIR, any difference was erased through the use of the Reg Refine tool. This is illustrated in Fig. 2 where the mean and max errors are plotted versus the number of Reg Refine locks used during the DIR process. As seen in the figure, the mean error declines steadily until reaching a plateau after nine Reg Refine locks have been used in combination. The max error behaves differently whereby Reg Refine shows little impact until certain locks are added. The remaining POPI phantoms (POPI_1, and 3‐6) were handled well by the initial deformable registration, with those incorporating a rigid registration along the base of the lung performing slightly better. Reg Refine was not able to noticeably improve the results for these phantoms where the median errors were already below 1 mm in magnitude prior to using the Reg Refine tool.

**Table 2:** Registration error for the POPI_2 phantom after several different iterations of DIR were performed. Iterations included initial rigid alignment to the apex and base of the lung, the use of the Reg Refine tool, and the addition of CBCT noise. Of note is the importance of the initial rigid alignment, the improvement made through the use of Reg Refine tool, and the insignificance of CBCT noise for this anatomical site.

*Registration*	*Mean*	*Median*	*Max*	%<2 mm
Apex	5.0	1.5	35.4	59
Base	2.8	1.2	20.0	75
w/ Reg Refine				
Apex	1.1	0.9	5.7	90
Base	1.2	0.9	5.4	88
w/ Noise				
LDT (σ=18)	2.8	1.1	25.0	73
LDT (σ=36)	2.7	1.1	19.7	77
LDT (σ=72)	2.7	1.2	19.4	78
LDT (σ=108)	2.7	1.2	22.0	76

**Table 3:** Registration error and standard deviations (SDs) for POPI phantoms 1 and 3–6. Reg Refine was ineffective at reducing the errors further.

*Registration*	*Mean*	*Median*	*Max*	%<2 mm
Apex	1.4±0.3	0.8±0.1	10.9±5.8	86.6±2.1
Base	1.2±0.2	0.9±0.1	8.9±5.0	88.4±3.2

As assessed in this study, noise had little effect on the overall registration process within the 4D lung at the 100+ landmark locations. This was determined by assessing DIR in the POPI_2 phantom after different levels of noise were added to its secondary dataset. The level of noise was varied by starting with the NPS profile of the “Low Dose Thorax” protocol and adjusting the parameter “$a_{1}$” of Eq. (1) until different noise magnitudes were reached. In each case, the NPS profile was converted into a 2D noise image and used to create pseudo‐CBCTs for the DIR process. As seen in Table 2, the error statics remain unchanged even at high levels of noise magnitude not expected in clinical images. Different CBCT protocols (“Pelvis” and “Pelvis Spotlight”) were also tried with similar results.

### B. Head and neck phantoms

**Figure 3:**
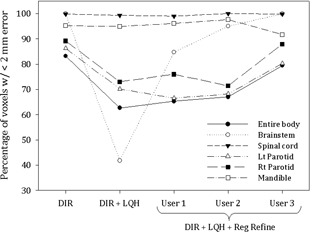
Registration error subdivided by DIR method and structure type. The data points represent the average of eight DIREP phantoms. Note the drop in registration accuracy after the application of low quality head (LQH) noise and the subsequent recovery within the brainstem through the use of the Reg Refine tool. User 3 was particularly successful at reducing registration errors in the parotids.

In contrast to the lung case, noise played a large role in determining the registration error in the head and neck. Table 4 highlights this effect for the DIREP_10 phantom. As seen in the table, each error statistic worsens when the noise associated with the “high quality head” protocol is added to the registration. The trend continues when the noise is switched from “high” to “standard” but then plateaus when switched from “standard” to “low”. The noise magnitude for each scan is also listed in Table 4 and mimics this trend. These effects were not the same across all contours. Noise greatly affected the registration accuracy of the brainstem, but had little effect on the spinal cord or mandible. The parotid glands were also affected but to a lesser degree. This can been seen visually in Fig. 3 where data from eight of the ten phantoms have been averaged then separated based on contour selection and type of registration performed. DIREP_5 was excluded due to technical issues. DIREP_9 was excluded as an outlier (7 mm mean error for the Rt Parotid compared to 0.5–1 mm for all other phantoms). The error statistic used for this plot is the number of voxels having a registration error less than 2 mm in magnitude. In the figure, the brainstem moves from 100% to 41% with an associated increase in median registration error, 0.4 (±0.2) to 2.7 (±1.5) mm. Conversely, the spinal cord and mandible remain constant near 99% and 95% with negligible changes in median error, 0.4 (±0.1) to 0.4 (±0.1) mm and 0.7 (±0.1) to 0.6 (±0.1) mm, respectively. The left and right parotid glands display an appreciable drop from 88% to 71% (averaged over both parotids) with their median registration error doubled, 0.7 (±0.3) to 0.6 (±0.2) mm increased to 0.5 (±0.3) and 1.3 (±0.2) mm.

Also shown in the figure is the impact of using Reg Refine to improve the accuracy of poor registrations. The data have been separated by user, and it can be seen that there is a clear distinction between User 3 and the other two users. While Users 1 and 2 were able to guide the system into making large improvements for the brainstem, only modest improvements were made for the parotid glands. User 3 significantly improved all contours not associated with bony anatomy. In cases where noise was not added, it was difficult to notice any improvement through the use of Reg Refine because the original deformable registrations were already very good. This was similar to what was seen with POPI phantoms 1 and 3–6.

**Table 4:** Registration error for the DIREP_10 phantom when different CBCT noise was added to the floating dataset during DIR. Note the dependence on noise magnitude and similarity in results when applying “standard quality head” and “low quality head” CBCT noise.

	*DIR w/Noise*			
	*DIR*	*High*	*Standard*	*Low*
Noise (σ)		18.5	38.7	40.3
Mean	2.1	3.1	3.7	3.7
Median	0.8	1.4	1.9	1.9
Max	30.5	31.4	30.5	30.7
%<2 mm	73.5	58.1	52.1	52.0

## IV. DISCUSSION

In this work, two aspects of deformable image registration were examined — the effect of CBCT noise on registration accuracy and the efficacy of the user guided tool Reg Refine. The former question was answered definitively for the MIM algorithm, considering that noise had no effect on registration error at the given points within the lung but played a large role in determining registration error within the head and neck. Contrast was plainly a determining factor with high contrasting structures, such as the lung, mandible, and vertebral bodies (surrogate of spinal cord), consistently maintaining high levels of accuracy regardless of the addition of CBCT noise. As previously noted, MIM incorporates an intensity‐based, free‐form (Demons) algorithm which utilizes multiple strategies to regularize the transformation. The minimization of bone deformation is one of these strategies, which helps explain the given results. In considering other anatomical sites where contrast between surrounding structures is far more muted (prostate, bladder, rectum, and abdomen), it is likely that CBCT noise would reduce the accuracy of deformable registration using the MIM algorithm. This would be similar to what was seen in a previous study which investigated both a Demons‐ and B‐spline‐based algorithm and found the Demons algorithm significantly more sensitive to noise, particularly in low‐contrast regions of the pelvis.^(9)^ Others have also noted this sensitivity and proposed a connection between the degrees of freedom (DOF) available to a DIR algorithm and the algorithm's response to noisy inputs.^(14)^ Since the current study focused exclusively on the MIM algorithm, only one half of this theory can be corroborated; that is the free‐form, high DOF algorithm evaluated in this work was sensitive to noise in low‐contrast scenarios. It is clear, however, that each deformable algorithm has unique characteristics which need to be assessed clinically before being implemented for a given use case.

The introduction of CBCT noise played an important role during the second part of this study. Because MIM adequately handled the majority of test cases available in the POPI and DIREP databases (out of 14 phantoms tested, only the POPI_2 phantom exhibited a median error > 1 mm when considering all landmarks/voxels), it was difficult to appreciate any benefit of using the Reg Refine tool. By adding CBCT noise, the baseline accuracy of the MIM algorithm was changed, thus providing a margin for improvement. Given this opportunity, Reg Refine was shown effective at reducing registration errors within the head and neck, particularly when controlled by a practiced user. Additionally for lung, Reg Refine was effective in the only case in which large errors existed.

**Figure 4:**
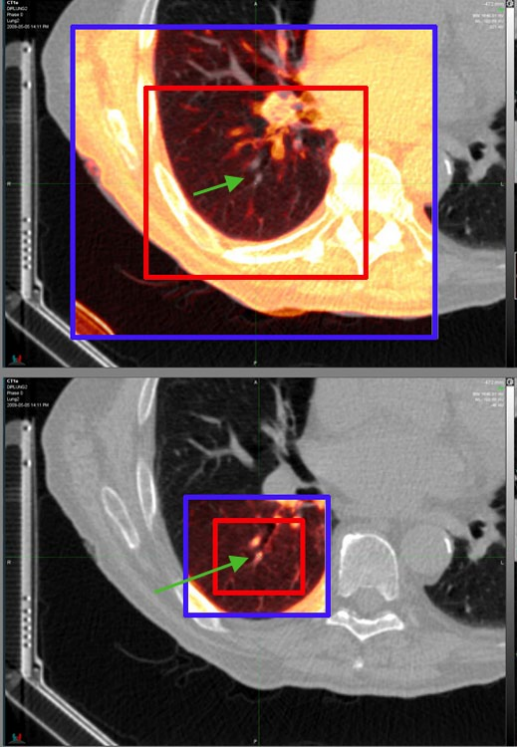
The application of Reg Refine within the 4D lung. The top image illustrates an incorrect usage of the tool where the rib has been included in the rigid registration window (red box). The lower image shows a much better alignment after the size of the window has been decreased. The green arrows denote to the local voxel which is the focus of the Reg Refine alignment. To better visualize the alignment between this voxel and its counterpart on the primary dataset, the registration is temporarily applied to every voxel within the blue box.

During the study, several strategies were identified which demonstrate how to best utilize the tool. When performing local box‐based rigid alignments, the amount of deformation in the nearby vicinity should be used to select the size of the registration box. Within the lung, where there is significant deformation everywhere, the size of the box should be kept small. An example of this is in trying to perform alignments along the outer edge of the lung where the ribs can move in a different direction than nearby lung tissue during respiration. If the registration box is set too large, the MIM algorithm will actively attempt to rigidly align the rib, thus misaligning the point of interest within the lung (Fig. 4). Vice versa, if the box is kept small, the MIM algorithm will not “see” the rib and only consider those tissues immediate to the point of interest which assumingly move rigidly with it. The opposite case exists when considering the brainstem. Here, the amount of deformation in the nearby vicinity is low, and the box should be opened widely to include the skull. The rigid registration of the skull acts a surrogate for the point of interest within the brainstem and is responsible for the large gains seen in this study with respect to this organ.

**Figure 5:**
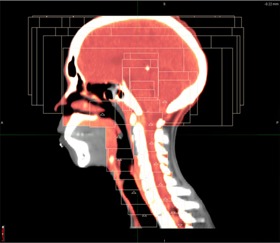
The application of the Reg Refine tool in the head and neck. Locks have been added along the spinal canal, within the cranium, along the trachea, and at the edges of the parotid glands.

The registration of the brainstem highlights both the power and limitation of the Reg Refine tool. In order to use the tool effectively the user must know, or at least have a good idea, how a given point on the floating dataset should be mapped to its corresponding location in the fixed dataset. In many cases the landscape is ambiguous, making any attempt to apply Reg Refine pure speculation. In these cases, the user may actually do more harm than good. It is important then during boxed‐based or manual alignment to focus on features such as edges, closed boundaries, and high contrasting structures which are known to have little deformation. Examples for the lung include borders around the pericardium, liver, and diaphragm, and also visible airways within the lung itself which can be matched locally based on shape. For the head and neck, the vertebral bodies are an excellent surrogate for the cord and should be aligned at several locations along the spinal canal (Fig. 5). Vessels and calcifications are also distinguishable landmarks which can be used to help guide the deformation. The air/tissue interface of the oropharynx is another feature to consider. For this structure there is less deformation posteriorly, making it is easier to envision how this interface should be aligned using the Reg Refine tool. The anterior and left/right interfaces may also be matched, though caution is warranted in cases where swallowing or mandible position cause significant changes in the local anatomy. For the parotids, the most effective approach was to apply five to seven Reg Refine locks, starting superiorly and focusing on the lateral border, aligning the parotids manually.

**Figure 6:**
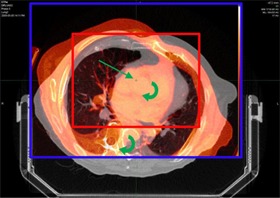
The application of the Reg Refine tool to correct implausible alignments. In this case, a large rotation would appear unlikely at the given location within the ascending aorta. The alignment at the voxel of interest (green arrow) has been applied to every voxel within the blue box to illustration the relationship between the given voxel and its counterpart on the primary dataset. Seeing the rotation in the spine helps the user understand that there is a significant rotation occurring at the point of interest.

In some instances, while the user may not know exactly how a given voxel is to be registered, it is clear that the current link between the fixed and floating datasets at the specified location is incorrect (i.e., there is no right answer, but there is a wrong one). An example would be an implausible shift, angle of rotation, or swirling (Fig. 6). In these cases, the Reg Refine tool can be used to establish a more realistic relationship between the two datasets at the given point. If at first attempt a box‐based alignment continues to produce poor results, the size of the box should be increased and retried or a manual approach may be applied. Once the registration is improved, the size of the box should be decreased to consider only the local neighborhood of voxels, a situation similar to what was described previously for lung tissue near the ribs.

In terms of optimizing the quantity of Reg Refine locks, it was difficult to ascertain a true correlation without rerunning hundreds of deformable registrations. While this type of analysis was completed for POPI_2 (optimized at 5–10 locks), only empirical observations were made for the head and neck phantoms where a minimum of 15–20 locks appeared justified. This was certainly a limitation of this study, as was the small number of users who participated in performing the registrations. The primary findings of this work, however, were not dependent on these factors and leave room for further investigation.

In considering other limitations, there were additional aspects of CBCT imaging not addressed during the creation of pseudo‐CBCTs. These include artifacts such as cupping, ringing, streaking, and aliasing, and also the potential for change in CT number due to variation in photon scatter. While it stands to reason that artifacts would reduce the accuracy of DIR, the Reg Refine tool at least provides the user a way to mitigate certain errors, specifically those caused by local artifacts. Errors due to global artifacts, such as aliasing and CT number change, are more difficult to address. Changes of 50–200 HU can be seen in comparing CT to CBCT images,^(15)^ and thus any algorithm applying a sum of squared difference similarity metric, such as MIM for CT‐CT DIR, has the potential to be affected. The overall magnitude of these effects should be quantified in future work.

## V. CONCLUSIONS

Unlike rigid registration, which can be assessed visually and controlled manually, DIR is often a blind process where trust in the system must be established though previous class‐based validation. Conversely, the Reg Refine tool available in MIM Maestro provides the user a tangible way to guide and evaluate the DIR process. It is unique in this regard amongst other commercial platforms. The tool has been shown effective at reducing registration error when controlled by an experienced user within the context of large lung deformation and CT–CBCT alignment of the head and neck. The latter case is important for the future of adaptive planning where CBCT noise has been shown to degrade the accuracy of DIR for low contrasting structures, such as the parotid glands. By effectively using the Reg Refine tool, these errors can be mitigated and reduced to levels previously associated with pure CT–CT alignment.

## COPYRIGHT

This work is licensed under a Creative Commons Attribution 4.0 International License.

## Supporting information

Supplementary Material
