# Erratum: Evaluation of the tool “Reg Refine” for user‐guided deformable image registration

**DOI:** 10.1120/jacmp.v17i5.6526

**Published:** 2016-09-08

## ORIGINAL CITATION

Perry B. Johnson, Kyle R. Padgett, Kuan L. Chen, Nesrin Dogan. Evaluation of the tool “Reg Refine” for user‐guided deformable image registration. J Appl Clin Med Phys. 2016;17(3):158�70.

Based on further information provided by the developers of the MIM Maestro algorithm, we would like to correct the following statements:

The DIR algorithm described in the manuscript is referred to as a “freeform (Demons‐based) transformation.” The MIM Maestro algorithm referenced is indeed a freeform DIR, but does not derive from the Demons algorithm.

In Step 2 of the Reg Refine process, it was stated that “all the vectors pointing from one dataset to the next are identical”. The statement should have read that “all the vectors are identical in magnitude” as opposed to implying they are identical in both magnitude and direction.

In Step 3 of the Reg Refine process, it was stated that the “rigid registration changes based on the deformation vector found at the center voxel.” The process is slightly more complicated, whereby a sampling of a small 15×15×15 mm area around the center of the visualization window is used to determine the best rigid transform approximation, in a least squared errors sense, to the deformations within this sampling cube. This approximation is constrained such that the deformation vector at the center of the visualization window is exactly represented by the rigid transformation.

In Step 8 it was stated that the “lock effectively replaces the deformation vector in the initial DVF” and is used “as improved initial conditions during a second deformable registration”. In fact, these user‐guided rigid registrations are considered in whole, not exclusively the modified vector at the center of the Reg Refine visualization window. Each user‐defined input or “locked” rigid registration is combined into a DVF, which is the Gaussian mix of all the local rigid registrations. Furthermore, this DVF is not only used as an initial condition, but the DIR algorithm also incorporates a bias term to guide the final deformation closer to the user defined local rigid registrations.

We thank the vendor for helping to clarify these statements.

