# Supplementary figures and images for: Evaluation of the tool “Reg Refine” for user‐guided deformable image registration
Source: J Appl Clin Med Phys. 2016 May 8;17(3):158–70. doi: 10.1120/jacmp.v17i3.6025 (PMC5690944; doi:10.1120/jacmp.v17i3.6025)

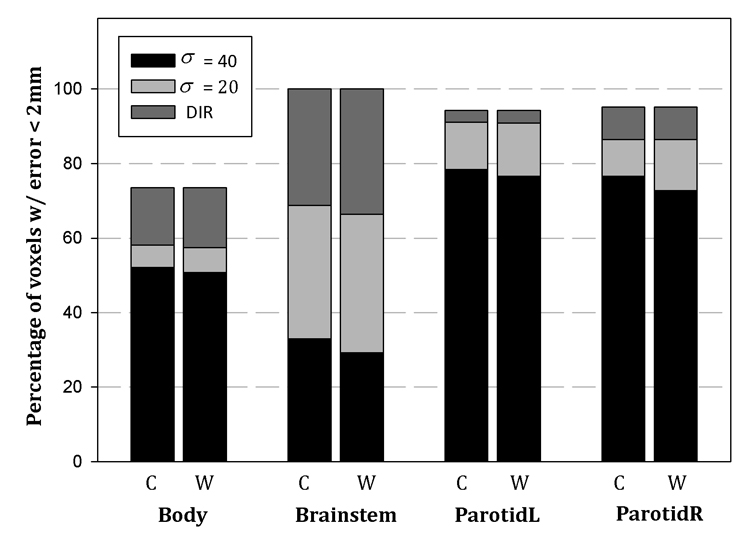

Supplement: Supplementary file 1 — Supplementary Material [file ACM2-17-158-s001.jpg]
